# Bilateral Sequential Non-arteritic Anterior Ischemic Optic Neuropathy (NAION)

**DOI:** 10.7759/cureus.19408

**Published:** 2021-11-09

**Authors:** Wang Shir Yen, Sugumar Yathavan, Muhamad Amin Ramli, Foo Siu Wan, Jemaima Che Hamzah

**Affiliations:** 1 Department of Ophthalmology, Faculty of Medicine, Universiti Kebangsaan Malaysia, Kuala Lumpur, MYS; 2 Department of Ophthalmology, Hospital Pulau Pinang, Ministry of Health Malaysia, George Town, MYS

**Keywords:** bilateral sequential naion, case report, tadalafil, phosphosdiesterase type 5 (pde 5) inhibitors, non-artertic anterior ischemic optic neuropathy (naion)

## Abstract

This is a case report of a rare case of bilateral sequential non-arteritic anterior ischemic optic neuropathy (NAION). A 50-year-old Indian gentleman, who is a known case of diabetes and an active smoker, presented with a right eye painless inferior visual field defect upon waking up from sleep. Fundoscopy revealed swollen right optic disc with peripapillary splinter hemorrhage while Humphrey visual field (HVF) showed right inferior altitudinal scotoma. Computed tomography of the brain and orbit proceeded to rule out compressive lesions. Thus, a diagnosis of right eye NAION was made. Three months later, he complained of a worsening visual field of the right eye. His right eye's optic disc was pale; however, the left was hyperemic and swollen with peripapillary splinter hemorrhage. HVF showed right eye tunnel vision while the left eye displayed inferior arcuate scotoma. Further investigation revealed suspicious enhancement of both intra-orbital optic nerves in magnetic resonance imaging suggestive of bilateral optic neuritis. Diagnosis of bilateral atypical optic neuritis was made. Thus, the patient was loaded with intravenous methylprednisolone 1 g/day for five days and subsequently oral steroid in tapering doses along with topical brimonidine tartrate 0.2%. Despite that, his left eye's visual field progressively deteriorated to inferior altitudinal scotoma. Subsequently, the lumbar puncture test performed was unremarkable while repeated MRI of the spine and brain showed no focal enhancing lesion. The patient revealed that he had a history of taking phosphodiesterase type 5 (PDE5) inhibitor (tadalafil) on and off over the past year. Diagnosis of bilateral sequential NAION was made and he was co-managed with the endocrine team to optimize his diabetic status. His subsequent visual field remained static with right eye tunnel vision and left eye inferior altitudinal scotoma. In conclusion, progressive NAION of the same eye or fellow eye is rare and mandates further investigations. It is important to identify and manage all the systemic and local risk factors to prevent further attacks. Although there is no rigid evidence stating that intake of PDE5 inhibitors can directly lead to NAION, patients with co-existing predisposing risk factors should be warned about possible ischemic ocular side effects of PDE5 inhibitors.

## Introduction

Non-arteritic anterior ischemic optic neuropathy (NAION) is the most common acute optic neuropathy in patients older than 50 years [1]. The estimated annual incidence of NAION among them is around 2.3 to 10.2 per 100,000 population, with about 1,500 to 6,000 new cases seen each year in the United States [2-4]. It is caused by hypoperfusion of the anterior portion of the optic disc supplied by short posterior ciliary arteries, which is influenced by both systemic and local factors. These include advanced age, hypertension, diabetes mellitus, hyperlipidemia, nocturnal hypotension, smoking, and small disc cup [5-6]. Due to the multifactorial nature of the disease, there is no effective therapeutic intervention to halt the progression of the disease in the same eye or the fellow eye. However, for the past 20 years, there were a few sporadic NAION cases in different countries that were reported to be associated with the usage of phosphodiesterase type 5 (PDE5) inhibitors [7-10]. This report presents a patient with predisposing factors and a history of consuming PDE5 inhibitors who presented with progressive bilateral NAION.

## Case presentation

**Figure 1:**
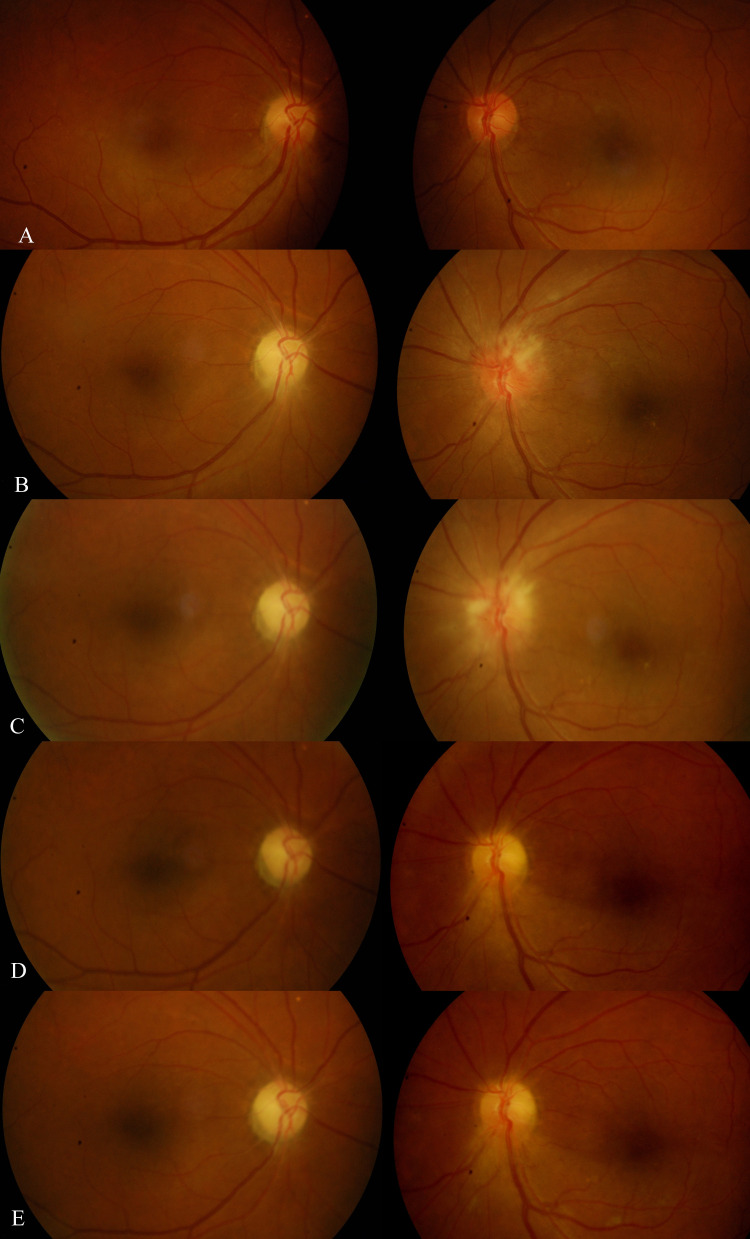
Serial color fundus photography. (A) Right eye's swollen optic disc while the left eye's optic disc is normal. (B)-(E) Right eye progressed to a pale optic disc while the left eye progressed to a swollen optic disc and subsequently regression of swelling with a pale superior half optic disc.

**Figure 2:**
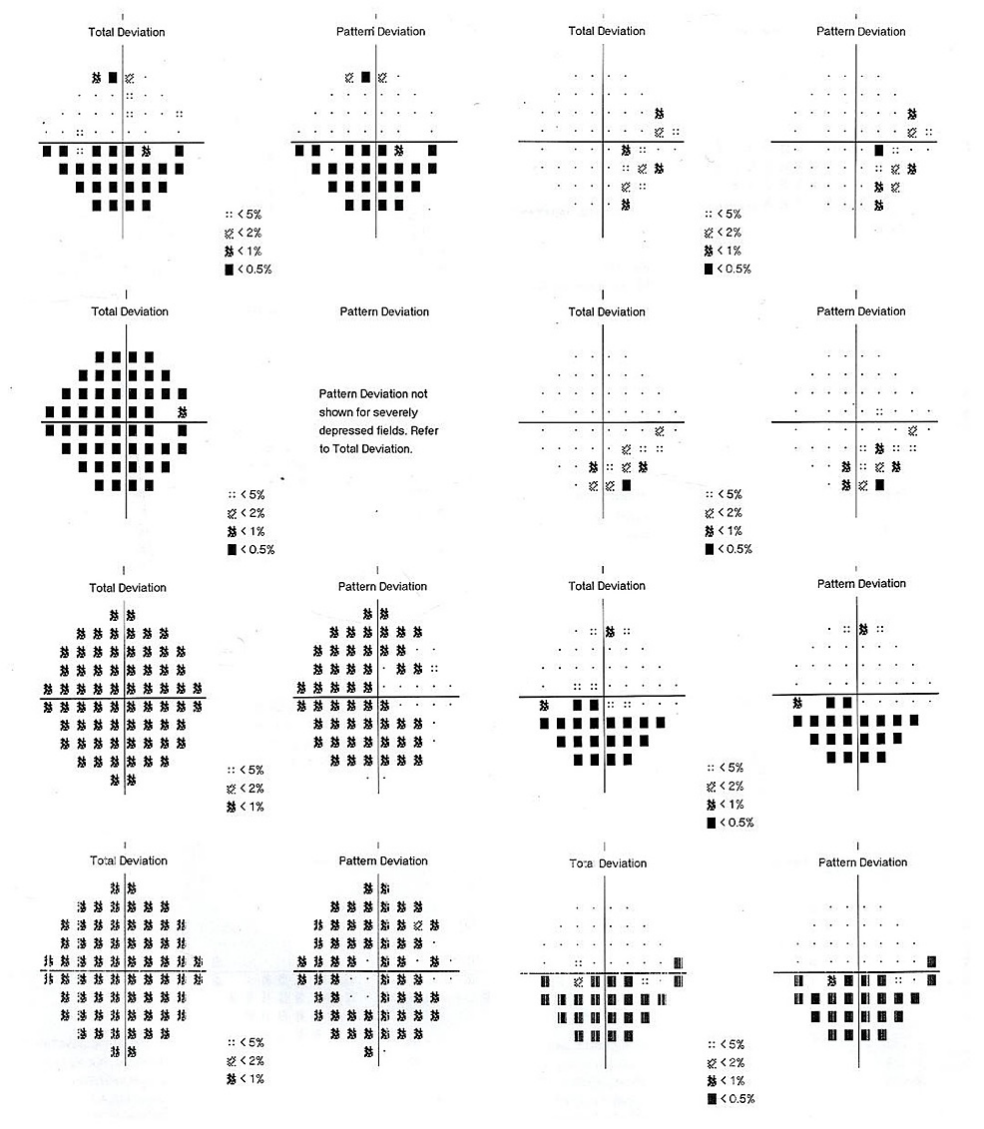
HVF shows right eye progressed from inferior altitudinal scotoma to tunnel vision while left eye progressed to inferior altitudinal scotoma. HVF, Humphrey visual field.

A 50-year-old Indian gentleman, a known case of diabetes and an active smoker, presented with a right eye painless inferior visual field defect upon waking up from sleep. At presentation, his right and left eyes' visual acuity (VA) were 6/24 and 6/9, respectively. Anterior segment examination was unremarkable in both eyes. Fundoscopy revealed swollen right optic disc with peripapillary splinter hemorrhage (Figure 1). Humphrey visual field (HVF) showed right inferior altitudinal scotoma. Computed tomography of the brain and orbit proceeded to rule out compressive lesions. Thus, a diagnosis of right eye NAION was made. Three months later, he complained of a worsening visual field of the right eye. VA remained static with the right eye (VA 6/24) and left eye (VA 6/9). Examination showed right eye relative afferent pupillary defect (RAPD) with impaired red saturation and light brightness. His right optic disc was pale; however, the left was hyperemic and swollen with peripapillary splinter hemorrhage (Figure 1). HVF showed right eye tunnel vision while the left eye displayed inferior arcuate scotoma (Figure 2). The patient was admitted for further investigations and was co-managed by the neuro-medical team. The visual evoked potential test was suggestive of right optic neuropathy. Serum glucose and serum hemoglobin A1c (HbA1c) levels were elevated, measuring 13.9 mmol/L and 9.1%, respectively. Serum total cholesterol (4.6 mmol/L) and low-density lipoprotein (LDL) cholesterol (2.1 mmol/L) were normal, but triglyceride level was high (3.3 mmol/L). Full blood count, erythrocyte sedimentation rate (2 mm/hour), and C-reactive protein (0.7 mg/dL) were all within normal limits. Serum anti-aquaporin-4, anti-nuclear antibodies, rheumatoid factor, and infective screening tests were negative. However, magnetic resonance imaging revealed suspicious enhancement of both intra-orbital optic nerves suggestive of bilateral optic neuritis. Diagnosis of bilateral atypical optic neuritis was made. The patient was loaded with intravenous methylprednisolone 1 g/day for five days and subsequently oral steroid in tapering doses along with topical brimonidine tartrate 0.2%. At the end of one month, his VA remained static and fundoscopy showed left eye resolving optic disc swelling. Despite that, his left eye's visual field progressively deteriorated to inferior altitudinal scotoma (Figure 2). Subsequently, we performed a lumbar puncture test, and the result was unremarkable. Repeated MRI of the spine and brain showed no focal enhancing lesion. Upon further history taking, the patient revealed that he had a history of taking phosphodiesterase type 5 (PDE5) inhibitor (tadalafil) on and off over the past year. Hence, we diagnosed him with bilateral sequential NAION, and he was co-managed with the endocrine team to optimize his diabetic status. His subsequent visual field remained static with right eye tunnel vision and left eye inferior altitudinal scotoma (Figure 2).

## Discussion

NAION is a multifactorial condition, with 72% of cases having pre-existing local or systemic risk factors [11]. These factors disrupt the optic disc blood supply’s autoregulation and cause an ischemic effect, although there is no reliable method to measure the posterior ciliary artery flow to the optic disc thus far. Among all, it is known that nocturnal hypotension is one of the most suggested risk factors, with 73% of cases reporting visual loss upon waking up from sleep [12]. It is postulated that PDE5 inhibitor, which is usually consumed at night, produces similar effects of nocturnal hypotension [13].

PDE5 inhibitors can disrupt autoregulation of optic disc via both local and systemic circulatory effects. It disrupts the nitric oxide (NO) balance in ocular circulation, which is essential in maintaining the basal vasodilator tone of ocular vessels [14]. Further damage is done by its systemic hypotensive effect on systolic and diastolic blood pressures. However, there are only a few cases reported in the literature that links NAION with the consumption of PDE5 inhibitors. This could be attributed to underreporting as patients are likely to be embarrassed to reveal the history of PDE5 inhibitor intake due to fear of social stigma, or they do not think it is relevant to their eye condition [15]. Diagnosis of NAION is challenging due to lack of confirmatory tests, especially when it has bilateral sequential involvement, as presented in this case. Bilateral NAION is extremely rare, with varied risk of fellow eye involvement ranging from around 15% to 25% based on different studies [11,16-17]. Male gender and those having systemic diseases such as diabetes are at higher risk of developing bilateral NAION [11]. As seen strongly in this case, our patient is at risk with systemic risk factors of being a male, diabetic, smoker, and has a history of consuming PDE5 inhibitors.

Due to the rare presentation of bilateral NAION, a low threshold of suspicion to rule out compressive and inflammatory causes of optic neuropathy is essential with appropriate imaging modalities. However, MRI findings can be non-specific, especially during the acute phase, as shown in a study by Rizzo et al. that compares optic neuritis with NAION whereby two out of 34 cases of NAION showed enhancement of optic nerves [18]. Despite no definite treatment for NAION, Hayreh et al. proved that the group given systemic steroids during the acute phase when optic disc edema is still present showed improvement in visual acuity and visual field compared to the untreated group [19]. Topical brimonidine has been proposed to have neuroprotective effects, although statistically, it has not been proven to improve visual acuity compared to placebo [20].

## Conclusions

Progressive NAION of the same eye or fellow eye is rare and mandates further investigations. Pathogenesis of NAION remains unclear with no definitive treatment for this condition. Thus, it is important to identify and manage all the systemic and local risk factors to prevent further attacks. There is no rigid evidence stating that intake of PDE5 inhibitors can directly lead to NAION. However, patients taking PDE5 inhibitors should be warned about possible ischemic ocular side effects especially those with co-existing predisposing risk factors such as diabetes mellitus.
